# Hindcasts and forecasts of suicide mortality in US: A modeling study

**DOI:** 10.1371/journal.pcbi.1010945

**Published:** 2023-03-13

**Authors:** Sasikiran Kandula, Mark Olfson, Madelyn S. Gould, Katherine M. Keyes, Jeffrey Shaman

**Affiliations:** 1 Department of Environmental Health Sciences, Columbia University, New York, New York, United States of America; 2 Department of Epidemiology, Columbia University, New York, New York, United States of America; 3 Department of Psychiatry, Columbia University, New York, New York, United States of America; Peking University, CHINA

## Abstract

Deaths by suicide, as well as suicidal ideations, plans and attempts, have been increasing in the US for the past two decades. Deployment of effective interventions would require timely, geographically well-resolved estimates of suicide activity. In this study, we evaluated the feasibility of a two-step process for predicting suicide mortality: a) generation of *hindcasts*, mortality estimates for past months for which observational data would not have been available if forecasts were generated in real-time; and b) generation of forecasts with observational data augmented with hindcasts. Calls to crisis hotline services and online queries to the Google search engine for suicide-related terms were used as proxy data sources to generate hindcasts. The primary hindcast model (*auto*) is an Autoregressive Integrated Moving average model (ARIMA), trained on suicide mortality rates alone. Three regression models augment hindcast estimates from *auto* with call rates (*calls*), GHT search rates (*ght*) and both datasets together (*calls_ght*). The 4 forecast models used are ARIMA models trained with corresponding hindcast estimates. All models were evaluated against a *baseline* random walk with drift model. Rolling monthly 6-month ahead forecasts for all 50 states between 2012 and 2020 were generated. Quantile score (QS) was used to assess the quality of the forecast distributions. Median QS for *auto* was better than *baseline* (0.114 vs. 0.21. Median QS of augmented models were lower than *auto*, but not significantly different from each other (Wilcoxon signed-rank test, *p* > .05). Forecasts from augmented models were also better calibrated. Together, these results provide evidence that proxy data can address delays in release of suicide mortality data and improve forecast quality. An operational forecast system of state-level suicide risk may be feasible with sustained engagement between modelers and public health departments to appraise data sources and methods as well as to continuously evaluate forecast accuracy.

## Author summary

Suicide deaths in the United States have increased considerably during the last two decades. Effective deployment of interventions can benefit from the availability of timely geographically well-resolved forecasts of suicide activity. Data from the National Vital Statistics System (NVSS), the most reliable source of mortality in the US, are released in yearly increments, thus constraining the ability of a forecast system reliant solely on this dataset, in generating timely estimates of current/future suicide activity.

In this study, we explored the feasibility of generating real-time monthly state-level forecasts of suicide deaths in the US. We augmented NVSS data with two sources of suicide-related behavior — calls to crisis hotline services and online queries to the Google search engine for suicide-related terms — and evaluated their utility as proxies to traditional surveillance systems.

Our results show that forecasts from standard autoregressive models improved over benchmark models; delays in release of suicide mortality data from traditional surveillance sources can be addressed in part using proxy data; and, besides providing more timely estimates of recent suicide mortality, proxy-based hindcast estimates improved forecast quality.

## Introduction

Deaths from suicide have risen for the last two decades in the United States [1,2]. Large-scale surveys have shown that besides deaths by suicide, a sizeable proportion of the population has suicidal thoughts (in 2020, 4.9 percent of adults 18 years or older; 12.2 million persons), and has planned (1.3%; 3.2 million) or attempted (0.5%; 1.2 million) suicide [3]. Several studies have quantified the differential effects of race, sex, and socioeconomic status on suicide risk and have documented higher risk among veterans and incarcerated populations [4–10]. Alongside these demographic attributes, geographical variability has also been noted with suicide mortality significantly higher in the midwest-, northwest and mountain states in the US [11].

In response, a broad range of preventive public health measures has been proposed and implemented. These encompass communication-oriented approaches to improve societal perception of suicide risk, reduce stigma, and promote help-seeking behavior, as well as measures to strengthen social connections, improve access to mental health services, and reduce access to lethal means [11,12].

While there is a significant body of research on associations between suicide mortality and individual and population level characteristics, and the relative efficacy of various public health interventions, predictive models of population risk remain relatively rare. Population level models can be useful for detecting changes in risk, either in overall trends or the emergence of suicide clusters, and can inform decisions on the timing, location and the type of interventions that are needed, or in evaluating interventions already in place. Previous studies have predominantly focused on national-level estimates in the US [13–15]. We believe that forecasts would be more actionable if they were generated with finer geographical resolution. For example, a forecasted increase in suicide deaths in a state can be used to trigger public health messaging about identifying warning signs, advertising of crisis hotline and other mental health services, and targeting of at-risk communities in that state. Therefore, we here explore the feasibility of generating real-time monthly forecasts of suicide deaths in each of the fifty states in the US.

A critical requirement for generating reliable forecasts is the availability of good quality, timely data, ideally covering a range of suicide behaviors from thoughts to deaths. However, accessing these data in real time remains challenging [12]. To focus on suicide deaths, the primary outcome in this study, the most reliable source of mortality in the US is the National Vital Statistics System (NVSS). Deaths reported to NVSS are released in yearly increments, resulting in a lag in availability of 11 to 23 months. Although the importance of accurate and robust surveillance estimates is indisputable, a forecast system that is reliant solely on this dataset would be constrained in generating timely forecasts.

An important contribution of this study is an evaluation of two proxy sources of suicide-related behavior to address the data lag in traditional surveillance systems. Crisis hotline telephone services, one of the data sources used here, connect individuals to crisis counselors and are a critical resource to those at risk of suicide. Call volumes to crisis call centers have increased over the last two decades and multiple studies indicate their effectiveness, with callers self-reporting fewer mental health crises or suicidal states in follow-up assessments [16–18]. Similarly, researchers have hypothesized that online activity in the aggregate, such as social media use, access of suicide-seeking and suicide-prevention websites, or related queries to search engines can predict suicidal ideation at a population level [19–23]. Here, we focused on query frequencies to the Google search engine for suicide and mental health related terms. Associations of suicide deaths with crisis calls and online searches are complex and under-studied with potentially large variability by location, time, and demographic characteristics. This analysis was limited to evaluating the utility of these data sources as predictive features in a forecast system and does not attempt to elucidate causal processes.

Using standard time series modeling methods and a combination of traditional and alternative data sources, we generated retrospective rolling monthly forecasts over nine years (2012-2020) for each of the 50 states in the US. This process included an intermediate step of generating *hindcast* estimates of suicide mortality, i.e. estimates for past months for which actual mortality data would not have been available if forecasts were generated in real-time. We report: a) the accuracy and reliability of the forecasts and intermediate hindcasts; b) the improvement in forecast quality from including hindcasts, overall and stratified by state and period; and, c) the relative difference in accuracy between forecasts and hindcasts from the two alternative sources of suicide-related activity.

## Materials and methods

### Suicide mortality rate

Records of all-cause deaths were obtained from the US National Vital Statistics System (NVSS) [24] and suicide deaths were identified using *International Classification of Diseases*, *Tenth Revision* underlying cause-of-death codes X60-X84, Y87.0, and U03 [25]. Monthly suicide counts in each state were calculated using the decedent’s county of residence and month of death. Annual state population estimates were obtained from the Bridged-Race Intercensal (2005-2010) [26] and Postcensal (2011-2020) [27] datasets and were assumed to remain unchanged during a calendar year. Monthly suicide mortality rates were calculated as suicide deaths per 100,000 population.

### Crisis hotline call rates

The 988 Suicide and Crisis Lifeline [Lifeline; https://988lifeline.org/ ], a network of over 200 round-the-clock toll-free centers, is the primary hotline for suicidal crisis and emotional distress counseling services in the US, accessible by phone as well as chat/text. Logs of calls routed to Lifeline centers were used to estimate the date of each call and caller location (inferred from the first six digits of the phone number) and aggregated to calculate monthly state-level call volumes. As above, annual state populations were used to estimate call rates per 100,000 population. As call rates are not normally distributed, a log transformation was applied. Access to Lifeline call volumes is a possibility but does not currently exist.

### Google Health Trends (GHT) API

The GHT API provides estimates of the proportion of user sessions on the Google search engine that included a query for a specified term. These estimates can be stratified by geography (country, state, etc.) and time (month, week, or day), and a historical record since 2004 is available. To identify terms whose search frequency can predict suicide mortality rates, we relied on prior studies that identified 6 categories comprising 111 suicide-related terms — *suicide seeking* (e.g. commit suicide), *suicide prevention* (e.g. suicide hotline), *suicide neutral* (e.g. suicides), *mood and anxiety* (e.g. depressed), *psychosis* (e.g. delusion) and *stressor or trauma* (e.g. social isolation) [23,28–40] (see S1 Appendix for a list of terms, by category). For each state, monthly search rates for a term category, defined as the proportion of user sessions from the state during a month that included one or more terms in the category, were retrieved and logit transformed. This choice is in part motivated by previous analyses that estimated influenza from search rates and found a logit transformation on search rates useful due to an approximately linear relationship between predictor and response in the logit space [41–43].

Monthly Google Health Trends (GHT) data are available at the end of each month, with a lag of less than one week. Access to the data feed must be requested through Google (see *Data availability*).

### Forecast generation

All-cause public use and restricted use mortality datasets are released by NVSS in one-year increments, usually in December of the following year, resulting in a 11 to 23 month lag for monthly suicide mortality records. For example, mortality data for all of 2020 were released in December 2021, resulting in a lag of 11 months for deaths that occurred in December 2020 and a lag of 23 months for those in January 2020. Provisional mortality counts for certain causes [44] may be available sooner, with varying degrees of completeness.

Forecast generation at month *m* proceeds in two steps: a) generation of *hindcast* estimates of suicide mortality for the time period between month *m* and the last available real observation (at earliest *m-12*); and b) generation of 6-month ahead forecasts using observations and hindcast estimates up to month *m*. Formally, let *y*_*k*_ denote the observed suicide mortality rate in month *k*, *Y*_*k*_ the time series of rates up to month *k*, (*y*_1_,⋯,*y*_*k*_); ${\bar{y}}_{m-l}$ the hindcast estimate for month *m-l* generated at month *m* and ${\bar{Y}}_{l}$ the time series $\left({\bar{y}}_{m-l},\cdots ,{\bar{y}}_{m}\right)$. At month *m*, hindcast estimates for *l* past months were generated using *Y*_*k*_, and forecast estimates for *h* months in the future, ${{\hat{y}}_{m+1},\ldots ,\hat{y}}_{m+h}$, were generated using the time series (*Y*_*k*_, ${\bar{Y}}_{l}$). If the last available observation is for month *k*, *l = m-k-1*, and as noted above, 12 *≤ l ≤* 24 and 1 *≤ h ≤* 6

### Model specification

Models used in this study are primarily based on the Autoregressive Integrated Moving Average (ARIMA) [45,46] approach and are described in detail in Text A in S1 Text. The primary hindcast model (*auto*) was trained on the time series of suicide mortality rates alone, and included components to capture trend and seasonality, the latter modeled with Fourier terms. Three models augment hindcast estimates from *auto* by including call rates (*calls*), GHT search rates (*ght*) and both datasets together (*calls_ght*). All 4 models were evaluated against a *baseline* persistence model with no trend or seasonality components, but which accounts for the average change per time step (random walk with drift [47,48]).

The models used to generate 6-month ahead forecasts were identical to the *auto* model described above and differed only in the data used to train the models — *auto*, *calls*, *ght* and *calls_ght* forecasts were generated using respective hindcast estimates appended to observed mortality rates. Change in hindcast/forecast quality relative to the *baseline* can be interpreted as the effect of a more careful modeling of the characteristics of the time series, while the difference of the augmented models, *calls*, *ght* and *calls_ght*, relative to *auto* can be interpreted as the advantage from incorporating more timely surveillance proxies.

Implementation of all models are available in *R* [49] packages *forecast [50]* and *fable [48]*.

### Experimental setup

The models were trained independently for each state using data solely pertaining to that state. Monthly data from all three sources were available for years 2007-2020 for all 50 states in the US. Suicide mortality data for 2020, which were set aside as a test set, were not used in model selection or hyperparameter tuning. We defined 2012 through 2019 as the validation period and generated rolling forecasts beginning January 2012, incrementing the training window one month at a time, and using only (and all) the data that would have been available were the estimates generated in real time (Fig A in S1 Text). For example, to generate retrospective forecasts at the end of January 2012, suicide mortality data from January 2007 through December 2010, and call rates and search rates for January 2007 through January 2012 were used to first generate monthly hindcast estimates for January 2011 through January 2012, and these estimates appended to mortality observations in order to generate forecasts for months February 2012 through July 2012. Hindcast models at the end of February 2012, were retrained with one additional month’s call and search rates, and forecast models were retrained with the new hindcast estimates. This iterative process was terminated at the end of 2019.

Model performance is reported separately for the validation (2012-2019) and test periods (2020), the latter being a better measure of model performance as it was withheld from both models and modelers.

### Evaluation metrics

Forecast models were used to generate quantile estimates, ${\hat{y}}_{\alpha ,m+h}$, at 23 levels, α={0.01,0.025,0.05,0.1,0.15,…,0.95,0.975,0.99}, and the median estimate, ${\hat{y}}_{.5,m+h}$, was used as a point estimate. Probabilistic and point estimates for hindcasts, ${\bar{y}}_{\alpha ,m-l}$ and ${\bar{y}}_{.5,m-l}$ were defined analogously (see Text B in S1 Text).

Accuracy of forecast point estimates was evaluated with mean absolute proportionate error (MAPE), where the accuracy of forecasts generated at month *m* was calculated as ${MAPE\_F}_{m}=\frac{1}{h}\sum_{h}\frac{abs(y_{h}-{\hat{y}}_{h})}{y_{h}}$. Accuracy of probabilistic forecasts were evaluated using quantile score (QS), calculated as ${QS\_F}_{m}=\sum_{\alpha }{QS\_F}_{\alpha ,m}$, where

$${QS\_F}_{\alpha ,m}=2*\alpha *(y_{h}-{\hat{y}}_{\alpha ,h})*1(y_{h}\geq {\hat{y}}_{\alpha ,h})+2*(1-\alpha )*({\hat{y}}_{\alpha ,h}-y_{h})*1(y_{h}<{\hat{y}}_{\alpha ,h}),$$

and **1**() denotes the indicator function, and α denotes the quantile level [47,51]. MAPE and quantile score of hindcasts were calculated analogously.

Both metrics are non-negative and can be interpreted as penalty measures with a higher value indicating an inferior estimate; a value of 0 indicates a perfect estimate. Summary measures are reported by aggregating (mean) across states and/or years and months. For each pair of models, Wilcoxon signed rank test was used to assess whether the difference in model quality per each metric was statistically significant [52].

In order to calculate model performance relative to a reference model, a *relative* measure was calculated. Relative quantile score (RQS) of forecasts generated with model *A* relative to reference model *R* is given by:

$$RQS\_F=\frac{100}{\left|s|*|m\right|}\sum_{s,m}\frac{{QS\_F}_{s,m}^{A}-{QS\_F}_{s,m}^{R}}{{QS\_F}_{s,m}^{A}+{QS\_F}_{s,m}^{R}}$$

where *s* and *m* denote the state and month at which forecasts were generated, respectively. *RQS*_*F* has well-defined bounds of [–100, 100] and is computable when at least one of the two models is not perfect (*QS* > 0). A negative RQS indicates an improvement over reference model.

Calibration of the hindcast and forecast probabilistic estimates were assessed by inspecting observations against estimated quantile distributions (see Text C in S1 Text). These were visualized as probability plots and the deviation from the diagonal was interpreted as a measure of miscalibration [53].

## Results

**Fig 1:**
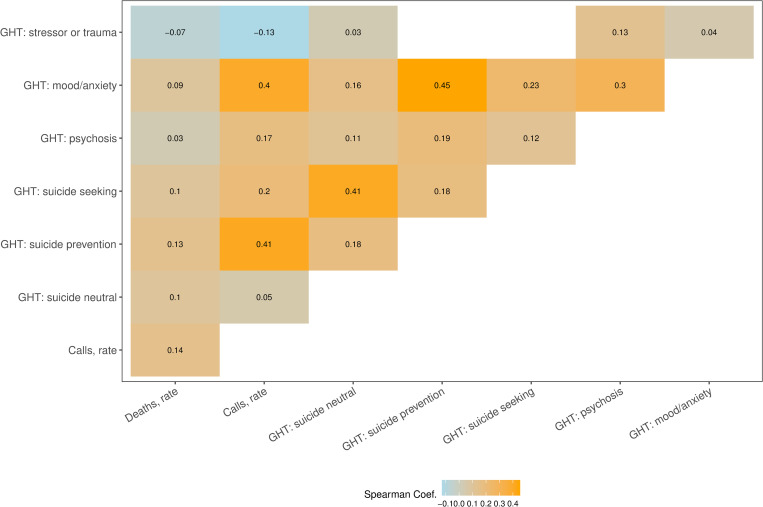
Pairwise Spearman correlation for each pair of variables. Correlations found to be not significant (*p* > .05) are not shown.

Fig 1 shows pairwise correlation between suicide mortality rates, call rates and six GHT search rates, estimated across all locations and over the entire study period (n = 8400; 50 locations * 168 months). All exogenous rates were found to have statistically significant, but small (|Spearman’s rho| < 0.15) correlations with mortality rates. Higher correlations were observed between call rates, and search rates for mood/anxiety (Spearman’s rho = 0.40, *p* < 1e-6) and suicide prevention (0.41, *p* < 1e-6) terms, as well as among search rate variables, particularly between mood/anxiety and suicide seeking (0.23, *p* < 1e-6), psychosis (0.3, *p* < 1e-6) and suicide prevention (0.45, *p* < 1e-6) terms.

### Validation period (2012-2019)

**Fig 2:**
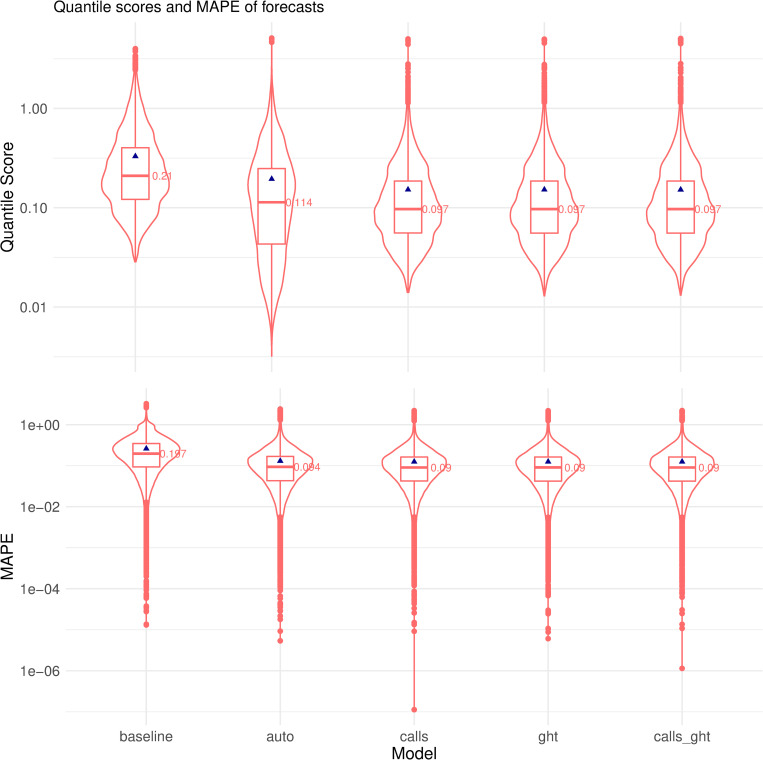
Boxplots of quantile scores and MAPE of forecasts from 5 models across all states and months. Blue points show mean estimate. *p*-value for Wilcoxon signed rank test on quantile scores: *calls*/*ght*=0.64; *calls*/*calls_ght* = 0.86; *ght*/*calls_ght* = 0.35. *p*-value for Wilcoxon signed rank test on MAPE: *calls*/*calls_ght* = 0.08; *ght*/*calls_ght* = 0.49. All other model pairs were statistically significant (*p* < 1e-4).

Fig 2 shows improved forecast quality when trend and seasonality in mortality rates were included in the models — the median quantile score for *auto* is lower than *baseline* (0.114 vs. 0.21), with an increase in spread (interquartile range: 0.28 vs. 0.21), and noticeably longer left tail indicating a higher proportion of good forecasts. Informing the models with near real-time proxy data further improved forecast quality. Augmented models *calls*, *ght* and *calls_ght* all had a lower median quantile score than *auto*, but their median scores were nearly indistinguishable from each other. This was further verified using two-sided Wilcoxon signed rank test where statistically significant differences (*p* < 1e-4) were observed between quantile scores of *auto* and *baseline* and between *auto* and each of the three augmented models, but not among any pair of augmented models (see S1 Text for results using MAPE as evaluation metric).

While the improvement in *auto* relative to *baseline* was also evident in the intermediate hindcasts, *calls*, *ght* and *calls_ght* were not significantly different from *auto* (Fig B in S1 Text). Hindcasts from *calls* were found to have better quantile scores than from the other two augmented models.

**Fig 3:**
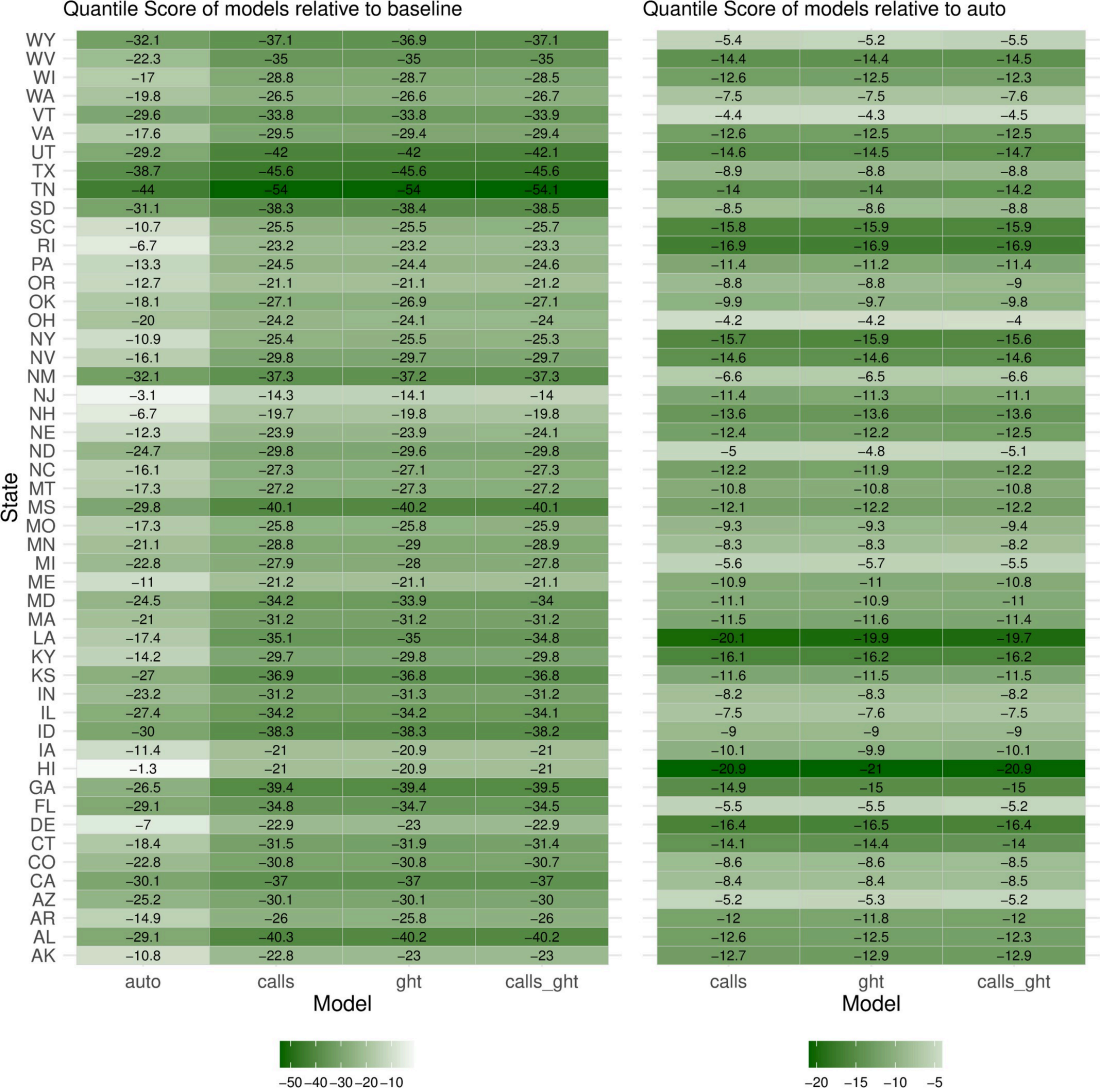
Quantile scores of forecasts from augmented models relative to *baseline* model (left) and relative to *auto* model (right), by state. The relative quantile score (RQS), has a range of -100 to 100, a negative value indicating a better forecast than the reference and a positive value indicating a worse forecast. The color lightness (light to dark) represents the magnitude of difference from reference, with a darker shade implying greater improvement.

Disaggregating forecasts by state showed consistent improvement of the augmented models’ quantile scores relative to both *baseline* and *auto* (RQS) across all states, with some variability in magnitude (Figs 3 and C in S1 Text for MAPE). In each state, RQS’ of the augmented models were nearly identical, suggesting little complementarity of the data sources and hence limited justification for their simultaneous inclusion in the models. This is in contrast to the differential state-wise improvement seen with hindcasts of the augmented models relative to *auto*, indicative of differences in predictive skill, and hence utility, of the data sources in generating hindcasts (Fig D in S1 Text).

**Fig 4:**
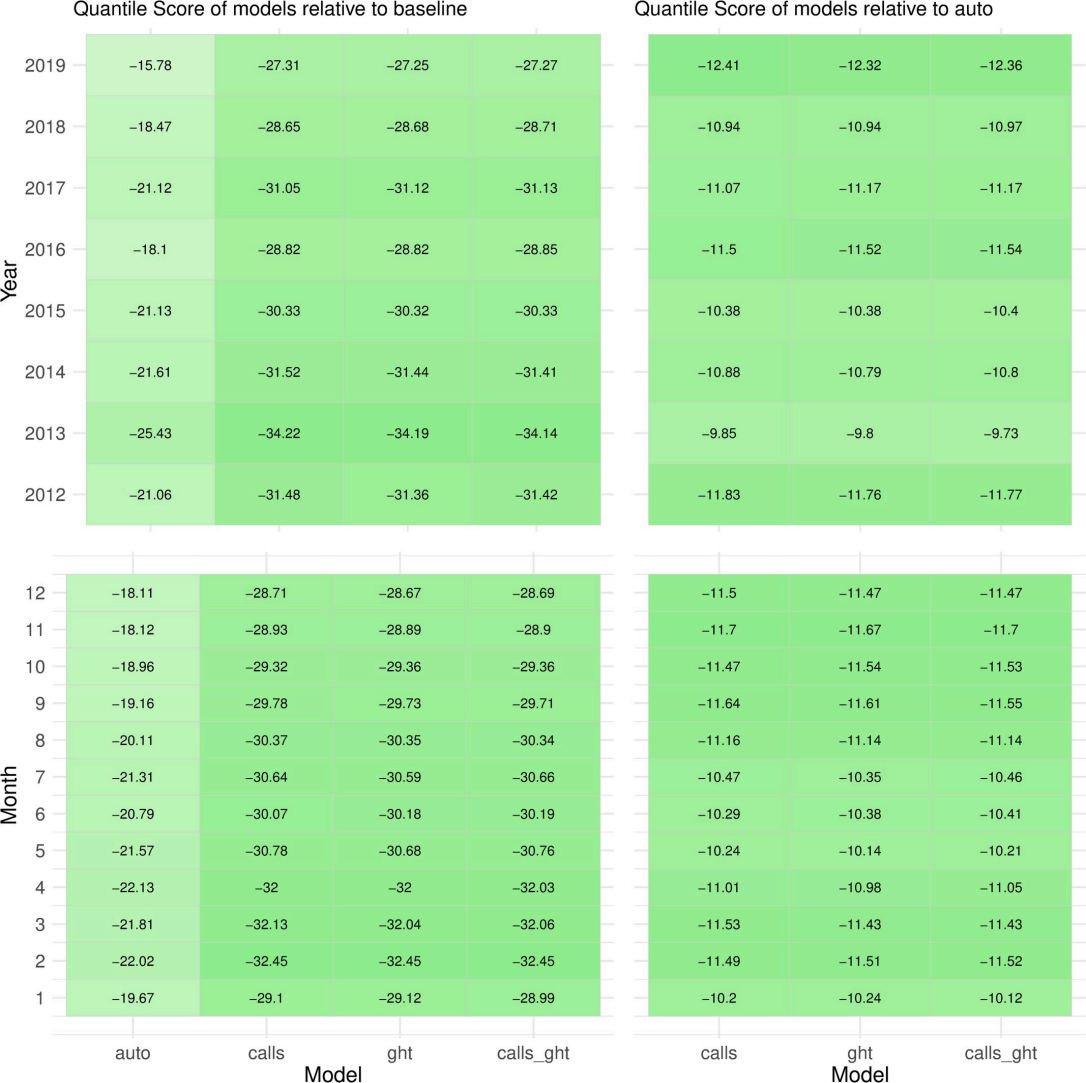
Quantile scores of forecasts from augmented models relative to *baseline* model (left column) and relative to *auto* model (right column), by year (top row) and month (bottom row). The relative quantile score (RQS), has a range of -100 to 100, a negative value indicating a better forecast than the reference and a positive value indicating a worse forecast. The color lightness (light to dark) represents the magnitude of difference from reference, with a darker shade implying greater improvement.

No clear difference in RQS was apparent when forecasts were stratified by month, with the possible exception of months May-July. A decrease in RQS of *auto* relative to *baseline* near the end of the validation period was also observed, possibly due to the plateauing of suicide mortality rates from 2018, leading to a disruption in the historical trend on which *auto* relies (Fig 4).

**Fig 5:**
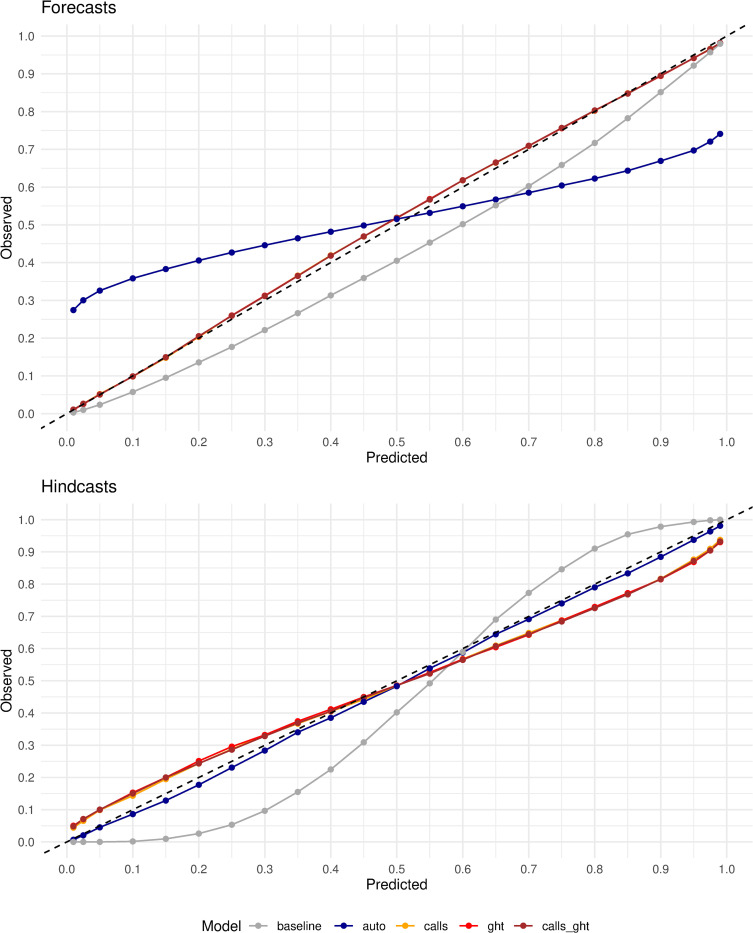
Calibration plot of forecasts (top) and hindcasts (bottom). Forecasts from *auto* (Cramer’s distance [53]=0.03) and to a lesser degree *baseline* (0.006) appear to be miscalibrated, while the remaining three models have similar and better calibration (5e-4). On the other hand, hindcasts from *auto* have the best calibration (7e-4) hindcasts and *baseline* the least calibrated (.015); the augmented models have similar, good calibration (3e-3).

Fig 5 demonstrates that hindcasts and forecasts from the augmented models have good calibration, with their corresponding probability plots nearly tracking the diagonal. While hindcasts from *auto* were also well-calibrated, their forecast distributions appear to have inadequate coverage for extreme instances of low and high mortality rates (also see Fig E in S1 Text). In contrast, both forecasts and hindcasts from *baseline* were miscalibrated, with hindcasts exhibiting a strong bias (forecasts skewed low/left) and a lack of sharpness.

### Test period (2020)

**Fig 6:**
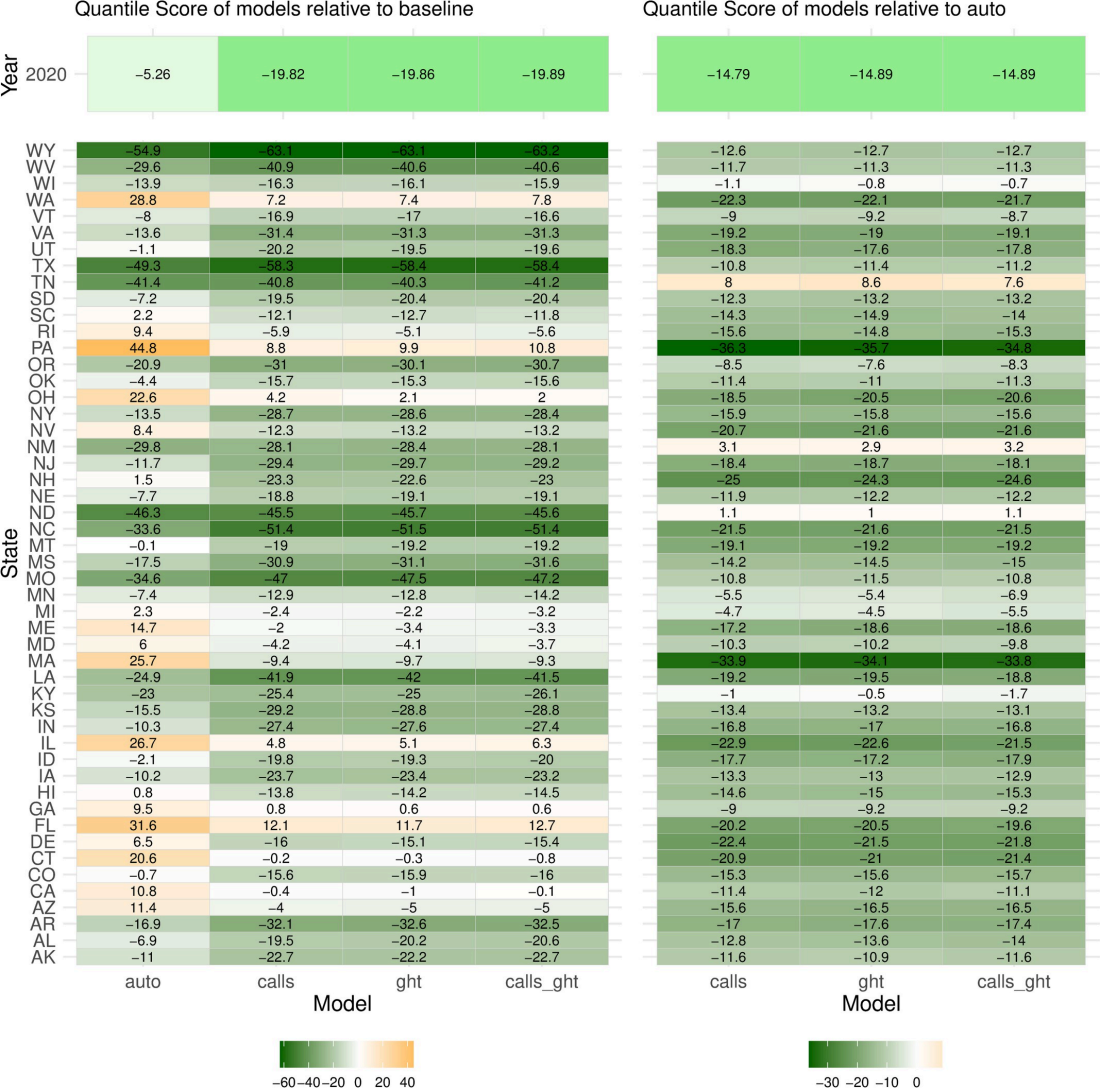
Quantile scores of forecasts from augmented models relative to *baseline* model (left) and relative to *auto* model (right), by state, during the test period (January 2020 – December 2020). Note that forecasts generated for the latter half of the year cannot be fully evaluated until mortality data for 2021 are available (for example, of forecasts generated at August 2020, 5-month ahead (Jan 2021) and 6-month ahead (Feb 2021) could not be evaluated).

Quantile score of forecasts and hindcasts from all models (except *baseline*) were higher for 2020 than in the validation period (Figs 6 and F in S1 Text). The largest deterioration in forecast quality occurred in the *auto* model, with the model underperforming *baseline* in 19 states. This is possibly an extension of the decrease in accuracy at the end of the validation period, due to lower suicide mortality beginning 2018. As seen in the calibration plots (Fig G in S1 Text), all models that included a trend component overestimated the mortality rate, with the miscalibration particularly evident for the *auto* model forecasts; augmented models continued to outperform the *baseline* and *auto* models.

### Multi-model ensembles

We evaluated the utility of an ensemble of models by averaging forecasts from three alternative methods to the fixed ARIMA model described above — a neural network based model, an exponential trend smoothing model, and a more flexible ARIMA approach that searches the parameter space at each month to identify best fit (Text D in S1 Text). During the validation period, the forecast quality of the ensemble was found to be not statistically different from the primary model used in this study. Encouragingly, however, during the test period two of the component models and the ensemble overall had better forecasts (statistically significant) than the fixed ARIMA model (Fig H in S1 Text). Calibration also improved.

## Discussion

This study aimed to evaluate the feasibility of generating monthly 6-month ahead forecasts of suicide mortality in US states. We have shown that forecasts from standard autoregressive models improved over benchmark models. We have also demonstrated that delays in release of suicide mortality data from traditional surveillance sources can be partially addressed by using proxy data, and inclusion of proxy-based hindcast estimates, besides providing more timely estimates of recent suicide mortality, improved forecast quality and rendered forecasts more sensitive to changes in long-term trends (as evidenced by performance during the 2020 test period). Forecasts from augmented models were also better calibrated even when their advantages, as assessed by aggregate measures of error, were less clear.

The choice of ARIMA as the primary model framework was motivated by our prior experience with this method, availability of robust implementations, relative conceptual simplicity and computational efficiency. While hyperparameter tuning and data transformations were handled to some extent by the *fable* software package, a more thorough exploration may further improve forecast quality. More recently developed time series modeling approaches, such as recurrent neural networks, have shown marked improvement in some domains [54,55], and their utility with the relatively short time series available here could be tested.

Although ARIMA-based forecast models can generate predictions at much longer horizons, the quality of the forecasts tends to degrade the farther ahead they project. The choice of a 6-month forecast horizon was believed to provide a reasonable trade-off between forecast quality and practical public health utility, and is in part influenced by forecast systems of influenza and other respiratory infections where a 4 time step horizon is commonly used.

The performance of the multi-model ensemble is in line with findings from prior studies in disease modeling [56–61] and other domains that ensembles often match or exceed individual model performance in the absence of a single reliably superior component model. Operationally, deploying an ensemble over a single model is likely to yield a more consistently good forecast quality. Modeling frameworks that capture suicide processes and mechanisms – thoughts, plans and attempts – would serve as valuable complements to the statistical models described here and need to be pursued as an important addition to ensembles [62,63].

Forecasts would be more actionable if they can be generated at sub-state resolutions, and/or tailored to specific population sub-groups (for example, young adults or marginalized communities). Models to flag emerging clusters among population subgroups would also be useful in deploying targeted interventions. Neither of the two data sources used in this study support stratifying by demographic attributes (such as age, sex and race/ethnicity), but aggregation at sub-state resolutions is possible, and reliability of such estimates remains to be investigated.

This study has several limitations. The models did not include socioeconomic or clinical predictors of mortality rate. We have described such models elsewhere [64] and note that the applicability of such methods would be contingent on the timely availability of covariate data. Similarly, better predictive models may be possible through inclusion of suicide-related information from neighboring states or the US overall rather than treating each state as an isolated entity. In addition, the precise physical locations of Lifeline callers were not available and our inference of location from caller area code could have introduced errors among mobile phone callers who relocated from their home state. Errors in mortality rate estimates are possible due to inconsistencies in death certification across states and study period, and potential undercounting of suicide deaths among certain racial/ethnic minority groups [65]. Additionally, the test period overlapped with the first year of the COVID-19 pandemic, and although the pandemic does not appear to have increased the annual suicide burden in most states [66], differences during some months, as well as a change in the relation between suicide mortality rates and call/search rates could have impacted the evaluation.

While the different skill metrics we report provide reasonable evidence of the utility of the proposed approach, a formal comparison with forecasts based on human expert judgement may be necessary to assess the value of automated approaches. Similarly, although we demonstrated the viability of a forecast system for state-level suicide mortality in a retrospective setting, there are challenges to operationalizing such a system. Lifeline call data are not public, and real-time access is uncertain. With the launch of a national suicide and mental health crisis number (988), nationwide call logs would be potentially warehoused in a central system, but no data sharing plans have been disclosed, possibly owing to confidentiality concerns. Prior studies have examined the possibility of using other auxiliary non-surveillance sources, such as social media posts with mixed results [19–22], to predict population-level suicide risk.

As illustrated by the complementarity of call and search rates for hindcast estimates, models built using multiple data sources may be better able to capture a wider range of intrinsic features and provide more resilience against data characteristics of a single source. While real-time population-level forecast systems of suicide deaths hold promise, they would benefit from access to multiple historical suicide-related datasets to train models and timely release of up-to-date data.

Our results indicate that a simple autoregressive model built solely on public mortality data can substantially improve over baseline estimation approaches at the state-level and possibly at finer geographic scales such as county or city. GHT data are free and relatively easy to access over some of the other sources noted above and can improve forecast quality. Selection of a modeling approach, identification and evaluation of data sources on suicide behavior, and appraisal of the tradeoffs between complexity/expense and forecast accuracy depend on the context in which the models are deployed and used, and can benefit from sustained engagement between modelers and public health departments.

## Supporting information

S1 Text Text A: Hindcasts and forecasts. Text B: Generating quantile distributions and assessing point and probabilistic forecasts. Text C: Assessing model calibration. Text D: Multi-model ensembles. Fig A. Schematic representation of the time periods covered by observations, hindcasts and forecasts. At the current time, all observational data available are used to train the hindcast model and estimate mortality for past months without mortality observational data. Forecast models are trained on a time series stitched together with both mortality data and hindcast estimates. Fig B. Boxplots of quantile score and MAPE of hindcasts from 5 models across all states and months. Blue points show mean estimate. *p*-value for Wilcoxon signed rank test on quantile scores: *auto*/*calls*=0.3*; auto*/*ght*=0.43; *auto*/*calls_ght* = 0.47; *ght*/*calls_ght* = 0.87. *p*-value for Wilcoxon signed rank test on MAPE: *auto*/*calls* = 0.23; *ght*/*calls_ght* = 0.52. All other model pairs are statistically significant (*p* < 1e-4). Fig C. MAPE of forecasts relative to the baseline model (left) and relative to the auto model built with no real-time proxy data (right). Fig D. Quantile scores of hindcasts from augmented models relative to *baseline* model (left) and relative to *auto* model (right). Fig E. Hindcasts and 6-month ahead forecasts for 2019 in California. Distribution shown for the first month in each subpanel is the hindcast estimate and the rest are forecasts. Fig F. Quantile scores of hindcasts from augmented models relative to *baseline* model (left) and relative to *auto* model (right), during the test period (January 2020 – December 2020). Fig G. Calibration plot for forecasts (top) and hindcasts (bottom), during the test period (January 2020 – December 2020). Fig H. Quantile scores of forecasts from ensembles of augmented models relative to *baseline* model (left) and relative to *auto* model (right), by state, during the test period (January 2020 – December 2020).
(DOCX)

S1 Appendix List of search terms used to query Google Health Trends API, by category. (CSV)

S1 Data All files are R datasets. Mortality.Rds: Monthly suicide deaths observed in each state during the study period. Nowcasts.Rds: Model hindcast estimates. Forecasts.Rds: Model forecast estimates.
(ZIP)
