# Supplementary material for: Hindcasts and forecasts of suicide mortality in US: A modeling study
Source: PLoS Comput Biol. 2023 Mar 13;19(3):e1010945. doi: 10.1371/journal.pcbi.1010945 (PMC10047563; doi:10.1371/journal.pcbi.1010945)
Supplement: S1 Text — Text A: Hindcasts and forecasts. Text B: Generating quantile distributions and assessing point and probabilistic forecasts. Text C: Assessing model calibration. Text D: Multi-model ensembles. Fig A. Schematic representation of the time periods covered by observations, hindcasts and forecasts. At the current time, all observational data available are used to train the hindcast model and estimate mortality for past months without mortality observational data. Forecast models are trained on a time series stitched together with both mortality data and hindcast estimates. Fig B. Boxplots of quantile score and MAPE of hindcasts from 5 models across all states and months. Blue points show mean estimate. p-value for Wilcoxon signed rank test on quantile scores: auto/calls=0.3; auto/ght=0.43; auto/calls_ght = 0.47; ght/calls_ght = 0.87. p-value for Wilcoxon signed rank test on MAPE: auto/calls = 0.23; ght/calls_ght = 0.52. All other model pairs are statistically significant (p < 1e-4). Fig C. MAPE of forecasts relative to the baseline model (left) and relative to the auto model built with no real-time proxy data (right). Fig D. Quantile scores of hindcasts from augmented models relative to baseline model (left) and relative to auto model (right). Fig E. Hindcasts and 6-month ahead forecasts for 2019 in California. Distribution shown for the first month in each subpanel is the hindcast estimate and the rest are forecasts. Fig F. Quantile scores of hindcasts from augmented models relative to baseline model (left) and relative to auto model (right), during the test period (January 2020 – December 2020). Fig G. Calibration plot for forecasts (top) and hindcasts (bottom), during the test period (January 2020 – December 2020). Fig H. Quantile scores of forecasts from ensembles of augmented models relative to baseline model (left) and relative to auto model (right), by state, during the test period (January 2020 – December 2020). (DOCX) [file pcbi.1010945.s001.docx]

*Supplementary material for*

**Hindcasts and Forecasts of Suicide Mortality in US: a modeling study**

Sasikiran Kandula*^1^, Mark Olfson^2,3^, Madelyn S. Gould^2,3^, Katherine M. Keyes^2^, Jeffrey Shaman*^1^

^1^ Department of Environmental Health Sciences, Columbia University, New York, New York. United States of America.

^2^ Department of Epidemiology, Columbia University, New York, New York. United States of America.

^3^ Department of Psychiatry, Columbia University, New York, New York. United States of America.

**Corresponding author*:

**Email:** [sk3542@cumc.columbia.edu](mailto:sk3542@cumc.columbia.edu); jls106@cumc.columbia.edu

**Keywords:** mental health; suicide; public health surveillance; crisis hotlines; time series models

**Text A: Hindcasts and forecasts**

*Hindcast estimates with* auto *model*

A non-seasonal autoregressive integrated moving average (ARIMA) model is generally specified with three parameters *p, d, q*, denoting the order of the autoregressive (AR), differencing and moving average (MA) terms, respectively. If the order is not specified, *fable* searches a predefined prior for AR and MA parameters (*p* = (0, 5), *q* = (0, 5)) and picks values that optimize the corrected Akaike’s Information Criterion. The optimal differencing (*d* = (0, 2)) is estimated using Kwiatkowski-Phillips-Schmidt-Shin (KPSS) test.

Seasonality was modeled with Fourier terms, a series of sine and cosine terms, to approximate seasonality. This is an alternative to using dummy variables as it requires fewer terms in the predictor. Monthly seasonality was modeled using 4 pairs of sine and cosine terms. A simple linear trend term was also included.

*Hindcast estimates with augmented models*

Augmented hindcast models (*calls*, *ght* and *calls-ght*) are simple linear regression models with *auto*’s hindcast estimate and proxy rates as explanatory variables -- *calls* model was specified with two predictors (call rates and *auto*’s hindcast), *ght* with 7 predictors (search rates for each of the 6 term categories as a separate predictor) and the combined *calls_ght* models with 8 predictors. As noted in the main text, log and logit transformations were applied to the calls and GHT predictors respectively.

*Hindcast and forecasts with* baseline *model*

With a random walk model, *h*-month ahead estimate generated at month *k* is very similar to the last known observation. The model performs reasonably well when the first order differenced time series is white noise. In cases where there is an overall trend in the model, as is the case with suicide mortality over the study period, an additional drift parameter is recommended. Here, we used the average change per time step up to month *m,* to capture trend. Therefore, the *baseline* model’s *h-*month ahead from month *k* is given by:

$$\hat{y}_{k+h}= y_{k}+h*\left( \frac{y_{k}-y_{1}}{k-1} \right)+\epsilon_{k}$$

where $\epsilon_{k}$ denotes random noise. This formulation was used to generate both forecasts and hindcasts, using the corresponding last available observation as would be available if the estimates were being generated in real-time.

**Text B: Generating quantile distributions and assessing point and probabilistic forecasts**

The distributional forecasts of models were captured using quantile estimates, $\hat{y}_{\alpha, m+h}$, at 23 levels, $\alpha=\{0.01, 0.025, 0.05, 0.1, 0.15,\ldots, 0.95, 0.975, 0.99\}$. These quantile estimates were simulated from the fit model using innovations sampled from a normal distribution with mean=0 and standard deviation given by$\sqrt{\frac{1}{T-K}\sum_{t=1}^{T} e_{t}^{2}}$, where *e* denotes ARIMA errors over the fit period, *K* is the number of parameters and *T* the number of observations used to fit the model.

MAPE is used to assess the accuracy of a point forecast (a single value), while quantile score assesses probabilistic forecasts (a distribution). Probabilistic forecasts and measures of their accuracy are recommended over point measures, but more commonly measures of point and probabilistic forecast quality are reported together. Quantile score is a proper scoring rule (encourages honest reporting of forecasts) and allows more detailed analysis of model accuracy through decomposition of the score into reliability, resolution and uncertainty.

**Text C: Assessing model calibration**

Calibration helps us assess whether, in the long run, an event occurs at the same probability as predicted by the model. In the case of distributional forecasts, a quantile forecast estimate $\hat{y}_{\alpha, m+h}$ implies that the true mortality for month *m+h* is expected to be lower than $\hat{y}_{\alpha, m+h}$, $(100*\alpha)$% of the time. As a measure of calibration, for each model, at each $\alpha$ level, we calculated the proportion of observations that were lower than the corresponding forecast estimate. For a perfectly calibrated model, the observed ($\hat{\alpha})$ and predicted proportions ($\alpha)$ would be identical at each of the 23 levels, i.e. a scatter plot of $\hat{\alpha}$ (*y*-axis) against $\alpha$ (*x*-axis) would have all data points along the diagonal (slope=1, intercept=0). Data points above the diagonal indicate an under prediction.

In Figure 5, the *auto* hindcast model (seasonality, trend and autocorrelation alone) is very well calibrated (blue line, bottom plot), the *auto* forecast model is poorly calibrated (blue line, upper plot). We can also infer the type of miscalibration -- the auto model forecasts appear to be quite often over predictions at smaller quantiles and under predictions at larger quantiles. The augmented models did not have this issue and both their hindcasts and forecasts are well-calibrated.

**Text D: Multi-model ensemble**

We built an equally-weighted ensemble forecast estimate using forecasts from 4 component models. If $\hat{y}_{\alpha, m+h}^{cmpt}$ is component model *cmpt*’s *h*-month ahead forecast at the $\alpha$ level generated at month *m*, the ensemble estimate is calculated as a simple mean over all component models’ corresponding quantile estimates, given as $\hat{y}_{\alpha, m+h}^{ens}= \frac{1}{\left| cmpt \right|}\sum_{cmpt} \hat{y}_{\alpha, m+h}^{cmpt}$. The component models, in addition to the fixed ARIMA model described, above are based on:

*Neural Network*

A single hidden layer feed-forward network with lagged suicide mortality rates as input was also used. The NNETAR function in *fable* fits a NNAR(*p*, *P*, *k*)_s_ model, where *k* is the number of nodes in the hidden layer, *p* is the number of non-seasonal lags, *s* is the seasonality and *P* is the number of seasonal lags. For example, if *p*=3, *P*=2 and *s*=12 (monthly seasonality), $y_{m}$ is modeled as a function of $y_{m-1}+ y_{m-2}+ y_{m-3}+y_{m-12}+ y_{m-24}$. By default, *P* is set to 1, *p* is selected so as to optimize AIC and *k* is about half the number of input terms.

The neural network was initialized with random weights and trained. 20 iterations, with different set of initial weights in each iteration, were used. To generate forecasts more than one time steps ahead, the one-step ahead forecast was used as an observation, and the process repeated

*Exponential Trend Smoothing (ETS)*

An ETS model is specified with three components, error, trend, and seasonality with each component specified by its own set of parameters. For example, the error term can be modeled as either additive or multiplicative; seasonal component can be modeled with different periodicity and value of smoothing parameters. For each component, *fable* implementation tests different parameter combinations and picks the one that optimizes log-likelihood. In this analysis, we did not perform additional hyperparameter selection or tuning instead relying on package defaults.

*Automated ARIMA*

While the primary ARIMA model used in the study allowed the non-seasonal parameters to be picked to optimize AIC, both seasonality and trend components were imposed. We further relaxed this specification and allowed the order of the corresponding seasonal components, P, D, Q to also be optimized per AIC. Predefined priors are *P* = (0, 2) and *Q* = (0, 2) and *D* = (0, 1).

**
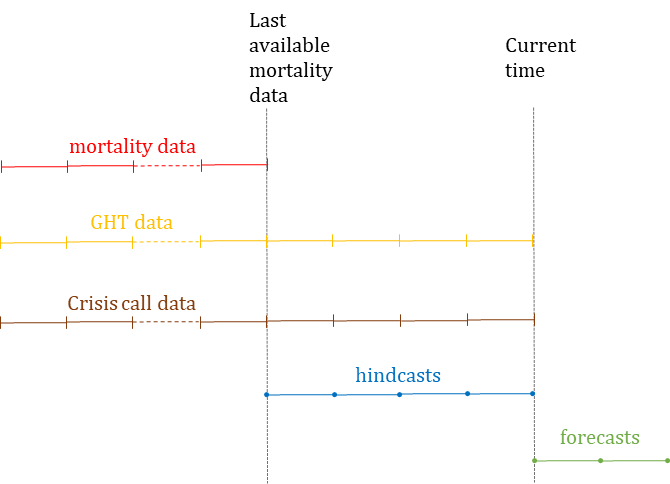
**

**Fig A.** Schematic representation of the time periods covered by observations, hindcasts and forecasts. At the current time, all observational data available are used to train the hindcast model and estimate mortality for past months without mortality observational data. Forecast models are trained on a time series stitched together with both mortality data and hindcast estimates

**
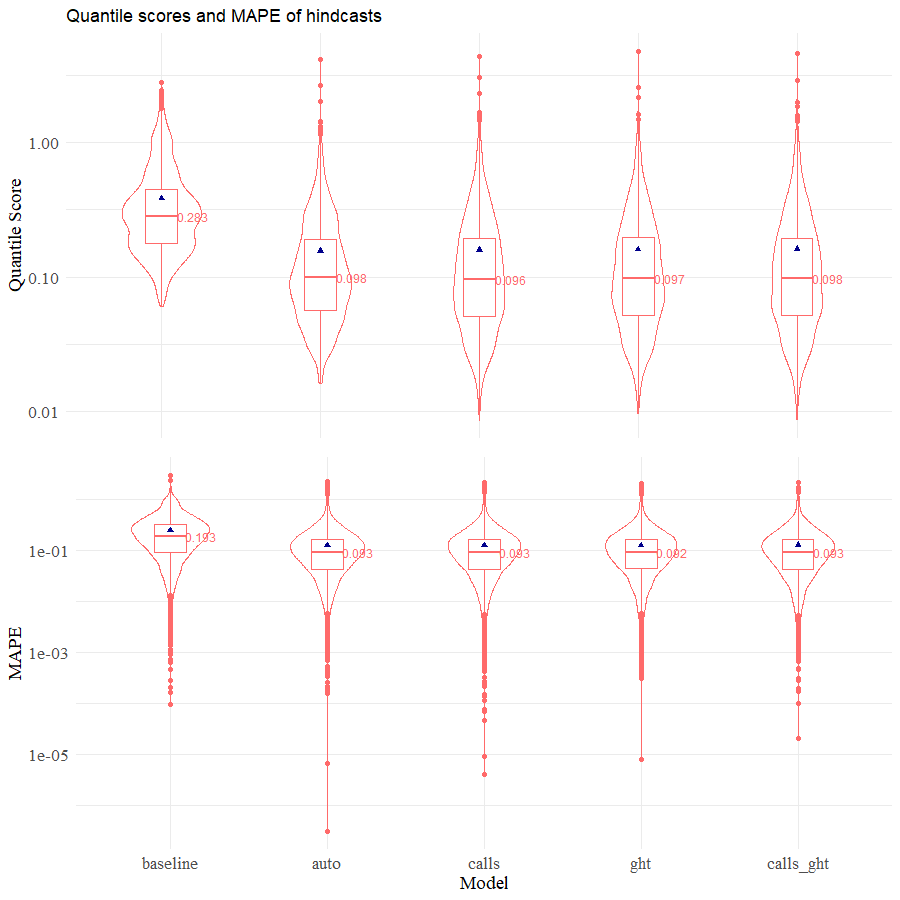
**

**Fig B.** Boxplots of quantile score and MAPE of hindcasts from 5 models across all states and months. Blue points show mean estimate. *p*-value for Wilcoxon signed rank test on quantile scores: *auto*/*calls*=0.3*; auto*/*ght*=0.43; *auto*/*calls_ght* = 0.47; *ght*/*calls_ght* = 0.87. *p*-value for Wilcoxon signed rank test on MAPE: *auto*/*calls* = 0.23; *ght*/*calls_ght* = 0.52. All other model pairs are statistically significant (*p* < 1e-4).

**
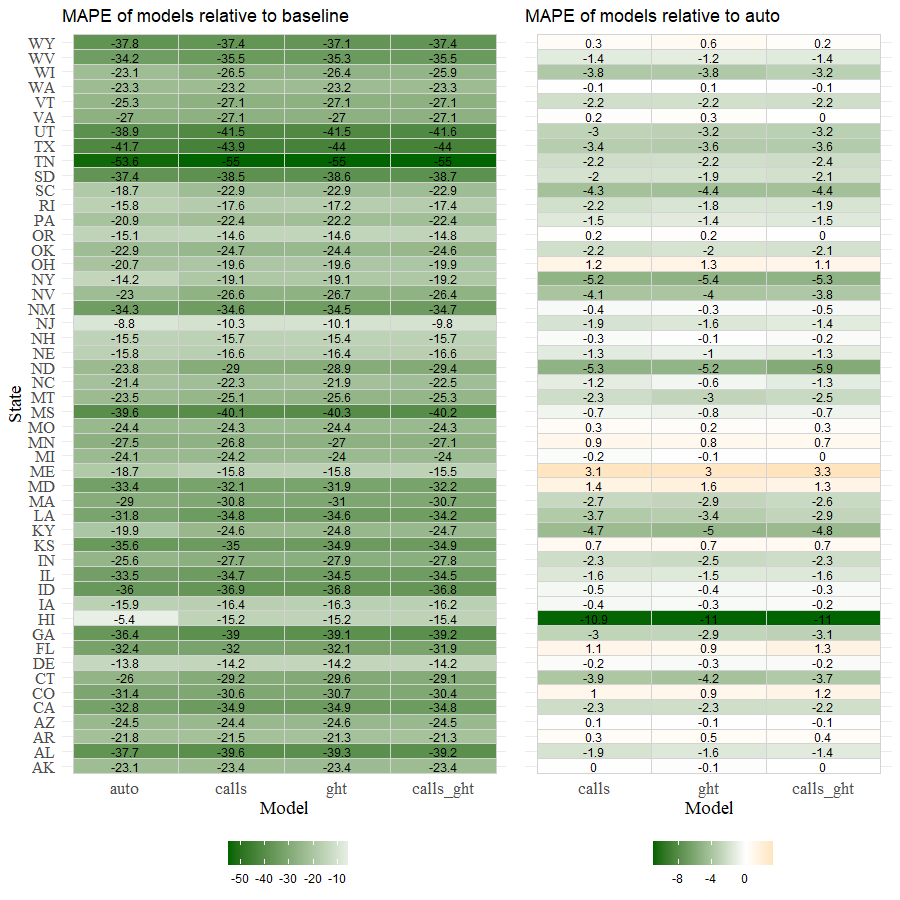
**

**Fig C.** MAPE of forecasts relative to the baseline model (left) and relative to the auto model built with no real-time proxy data (right)

**
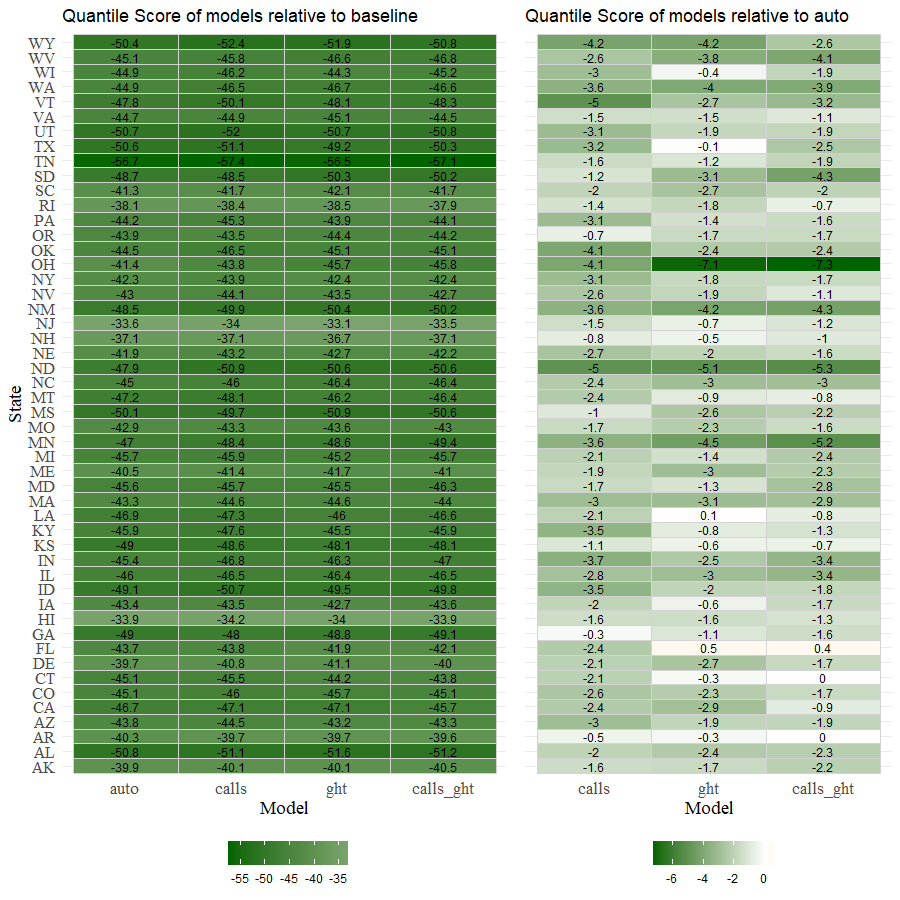
**

**Fig D.** Quantile scores of hindcasts from augmented models relative to *baseline* model (left) and relative to *auto* model (right).

**
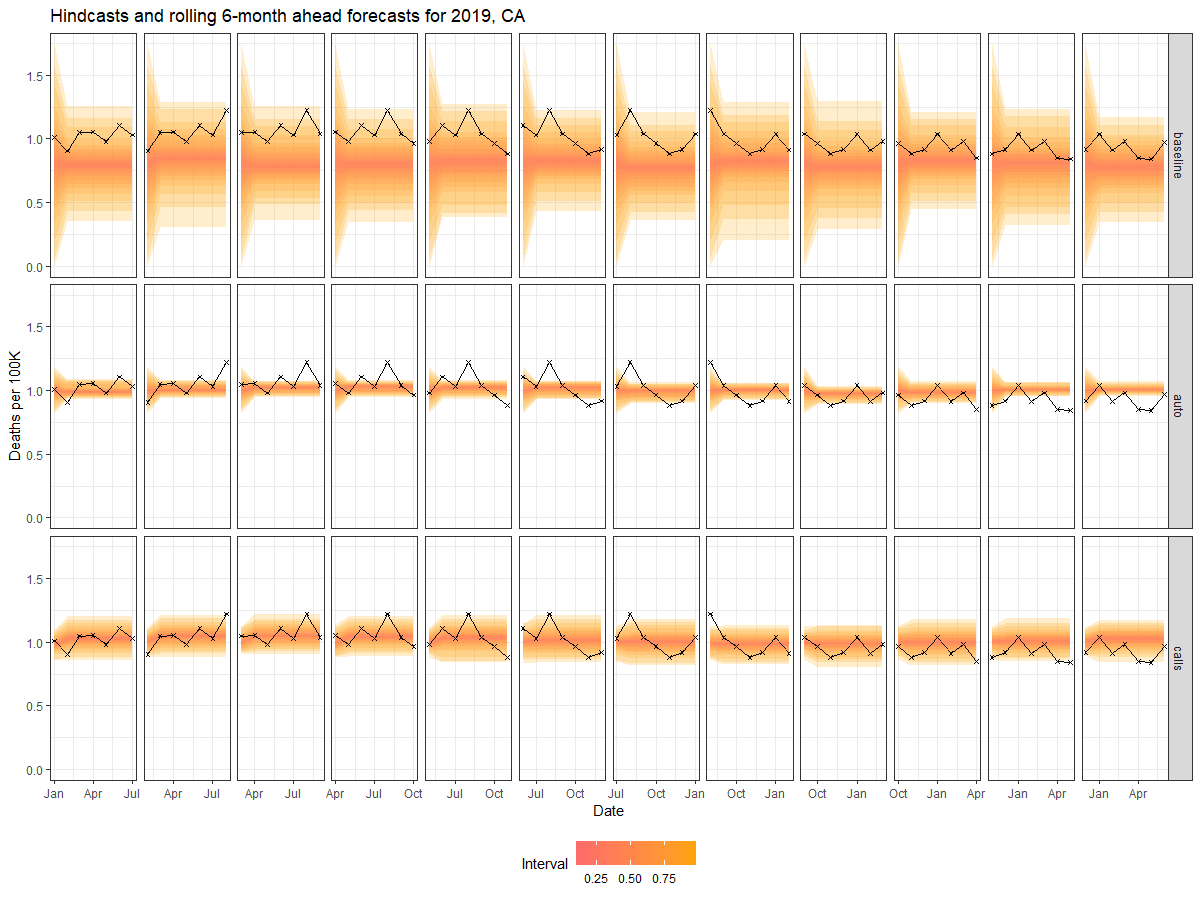
**

**Fig E.** Hindcasts and 6-month ahead forecasts for 2019 in California. Distribution shown for the first month in each subpanel is the hindcast estimate and the rest are forecasts.

**
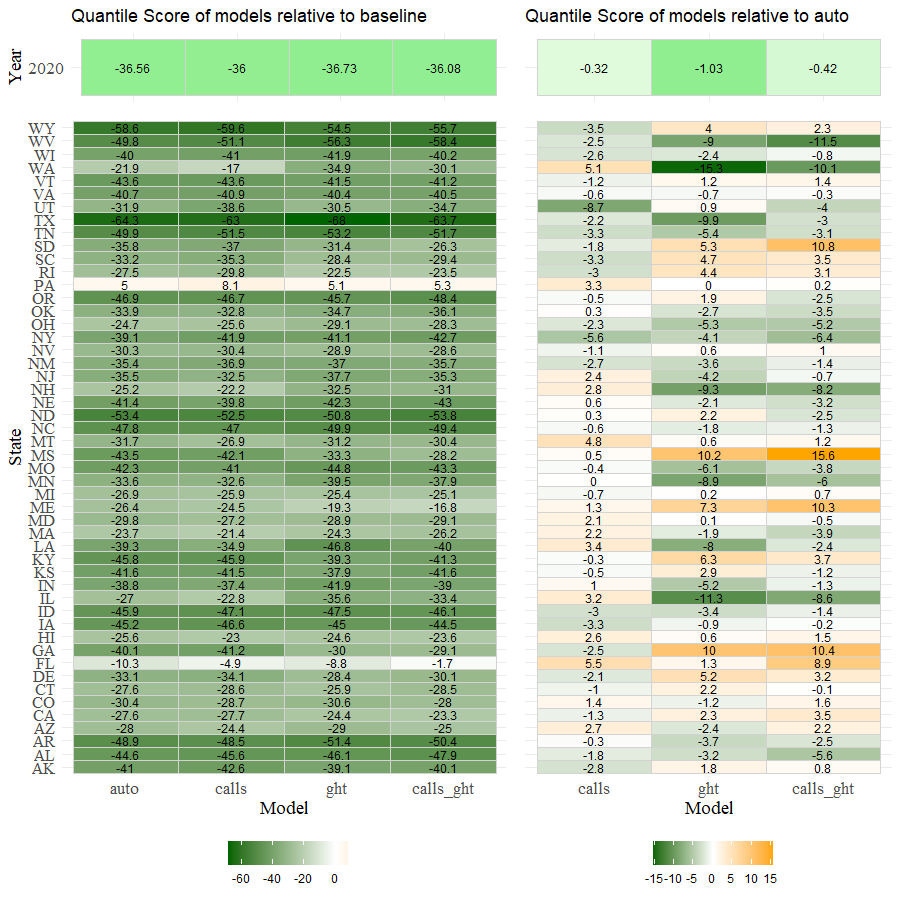
**

**Fig F.** Quantile scores of hindcasts from augmented models relative to *baseline* model (left) and relative to *auto* model (right), during the test period (January 2002 – December 2020).


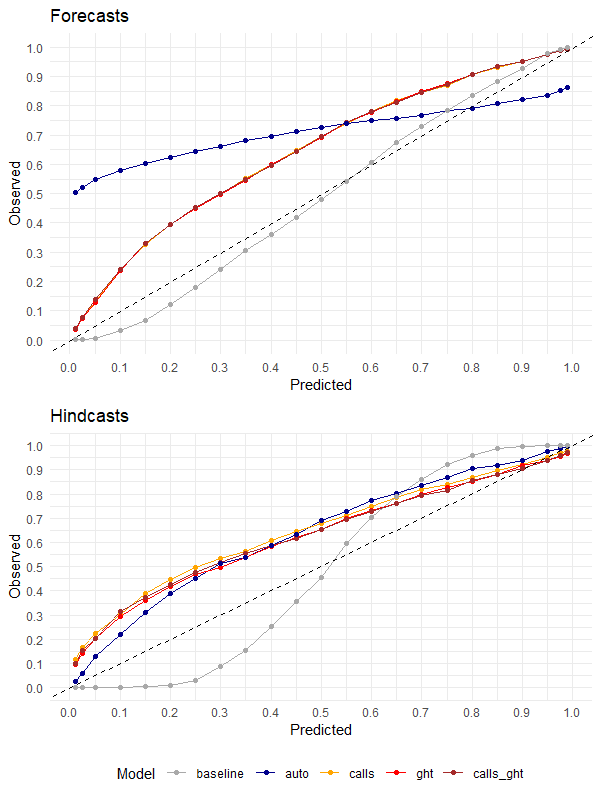


**Fig G.** Calibration plot for forecasts (top) and hindcasts (bottom) , during the test period (January 2002 – December 2020).

**
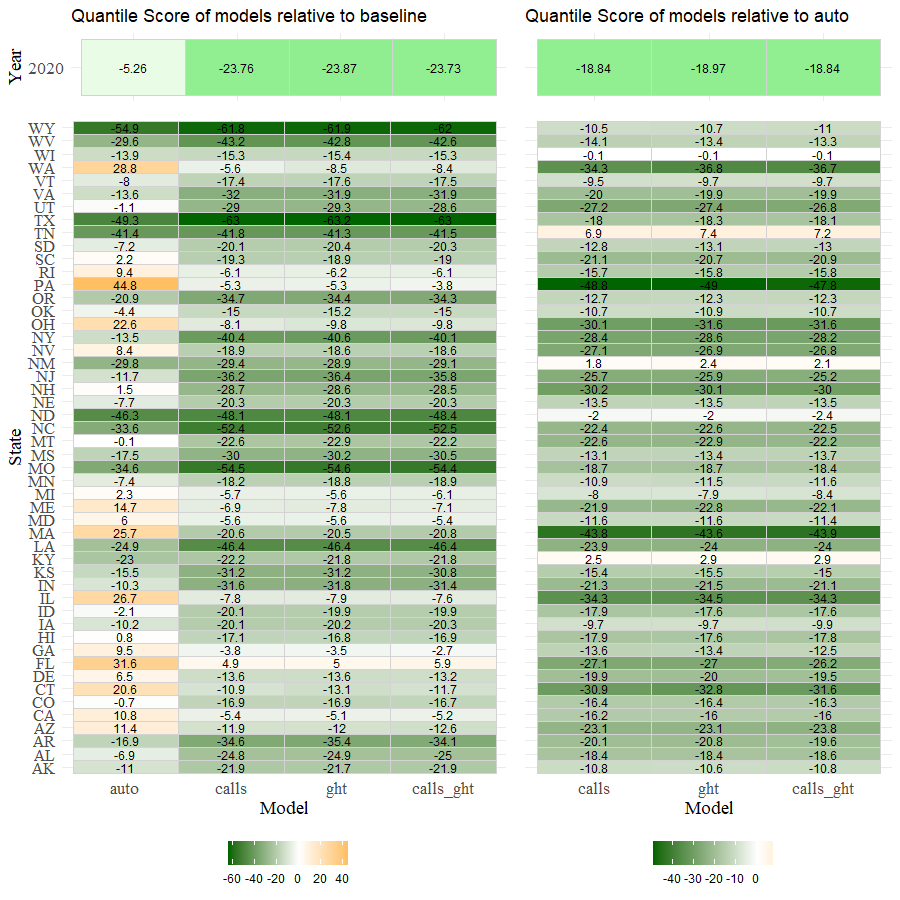
**

**Fig H.** Quantile scores of forecasts from ensembles of augmented models relative to *baseline* model (left) and relative to *auto* model (right), by state, during the test period (January 2020 – December 2020).

$$\vdots$$
